# A review of the integration of traditional, complementary and alternative medicine into the curriculum of South African medical schools

**DOI:** 10.1186/1472-6920-14-40

**Published:** 2014-02-28

**Authors:** Ethel Chitindingu, Gavin George, Jeff Gow

**Affiliations:** 1 Health Economics and HIV and AIDS Research Division (HEARD), University of KwaZulu-Natal, Durban, South Africa; 2 School of Commerce, University of Southern Queensland, Toowoomba, Australia

**Keywords:** Traditional, Complementary and alternative medicine, Medical schools, South Africa

## Abstract

**Background:**

Traditional, complementary and alternative (TCAM) medicine is consumed by a large majority of the South African population. In the context of increasing overall demand for healthcare this paper investigates the extent to which South African medical schools have incorporated TCAM into their curriculum because of the increased legislative and policy interest in formally incorporating TCAM into the health care system since democracy in 1994.

**Methods:**

Heads of School from seven South African medical schools were surveyed telephonically.

**Results:**

One school was teaching both Traditional African Medicine (TM) and CAM, five were teaching either TM or CAM and another was not teaching any aspect of TCAM.

**Conclusions:**

In conclusion, there is a paucity of curricula which incorporate TCAM. Medical schools have not responded to government policies or the contextual realities by incorporating TCAM into the curriculum for their students. South African medical schools need to review their curricula to increase their students’ knowledge of TCAM given the demands of the population and the legislative realities.

## Background

The use of traditional, complementary and alternative medicine (TCAM) is widespread in many countries of the world, specifically amongst patients with chronic or long term illnesses [1,2]. In Western societies more and more people have been using TCAM to treat their ailments, either as a complement or substitute to more bio-medical treatment regimens [3]. This has been due in part to the philosophical orientation of TCAM practitioners who actively engage with patients in their treatment which results in a more holistic approach to health [1,4]. Conventional Western bio-medicine is increasingly regarded as expensive, inaccessible, depersonalized and not completely effective, especially for those patients with chronic diseases [1].

Traditional medicine (TM) is separate from complementary and alternative medicine (CAM). According to the World Health Organization (WHO), TM is “health practices, approaches, knowledge and beliefs incorporating plant, animal and mineral-based medicines, spiritual therapies, manual techniques and exercises, applied singularly or in combination to maintain well-being, as well as to treat, diagnose or prevent illness” [5]. Hollenberg [6] defines CAM as “systems of health care distinct from bio-medicine that are practiced in rich “developed” nations and may or may not include systems or parts of TM”. Treatment practices which come under its heading include: Ayurvedic, Chinese medicine, Osteopathy, Chiropractic, Homeopathy, Naturopathy, Phototherapy, Unani-Tibb, Aromatherapy, Massage and Reflexology. However, there is provision in the Traditional Health Practitioners Act (Act 35 of 2004) to accommodate more practices [7].

There is much evidence of the increased use of TCAM in preference to bio-medicine [1,8]. This increase is confirmed by the billions of dollars constantly spent on TCAM therapies in Western countries [1,9]. It was estimated in the USA approximately 34 billion dollars per annum was spent on complimentary medicine in a 2007 survey [10]. This increase in spending is also found in African countries with households in Cameroon spending 7% of their income on traditional healing [7]. In South Africa, Nxumalo [11] found that 10% of household expenditure by the poor was on traditional healing. These levels of household expenditufre support other studies confirming high rates of use of TCAM in sub-Saharan Africa [6]. In South Africa in particular, 70 to 80% of black Africans use traditional medicine as the primary source of treatment for their health care needs [12]. Further, the use of traditional medicine is common amongst patients suffering from HIV/AIDS [12]. Various factors have been attributed to this shift including the close proximity of traditional healers to their communities and thus their widespread availability, especially in rural areas which are underserviced by bio-medicine [1]. The higher cost of conventional bio-medicine is a further factor resulting in increased usage of TCAM [8,9].

However, concern has been raised about the possible interference of TCAM with the pharmacology of conventional bio-medical drugs when used simultaneously which reduces the latter’s effectiveness [1]. TCAM is constantly discounted as a legitimate source of knowledge for health care by bio-medical practitioners because it does not follow the orthodox view of positivism [6]. Dantley [13] stresses, “The central assumption by which the culture of positivism rationalizes its position on theory and knowledge is the notion of objectivity”. This is contrary to TCAM methods which emphasizes the role of the individual in ‘solving’ their health care issues. Thus, there is a disjuncture between bio-medicine and TCAM.

The use of TCAM is increasing among many populations particularly those suffering from chronic illnesses. In some instances, bio-medical practitioners recommend TCAM to patients if they fail to respond to allopathic treatment. This global increase in demand for TCAM has led some medical schools in Western and many Asian countries to incorporate TCAM into their curricula [14].

This paper is the first review of the integration or otherwise of TCAM into the undergraduate and postgraduate medical curricula of South African medical schools within a context of increasing demand for TCAM services. Seven out of the eight South African medical schools agreed to participate in the study.

### Policy framework

In South Africa, TM and CAM are governed by two separate bodies. TM is regulated by the Traditional Healers Council (THC) and CAM by the Allied Health Professions Council of South Africa (AHPCSA). South Africa has made great strides to integrate TM and CAM into its health legislative framework [7]. The Traditional Health Practitioners’ Act of 2007 was passed to regulate TH practitioners. The main aim of the Act was to ensure “the efficacy, safety and control of traditional health care services, to provide for the management and control over the registration, training and conduct of practitioners, students…” [15]. In the Act, traditional health practice is defined as a practice enlisting indigenous African techniques and traditional medicine or practice principles [15]. In 2000, CAM and TM were declared in legislation to be on the same footing as bio-medicine. In 2007, approximately 200,000 traditional healers were registered with various bodies and 3600 complementary and alternative medical practitioners were registered with the AHPCSA [7]. These efforts signify a significant commitment by the South African government to regulate and authenticate TM and CAM and their practitioners.

Currently, the policy of the South African National Department of Health requires bio-medical doctors to serve in rural areas for two years following the completion of their training [16]. Given the black African bias of rural populations of over 90%, many bio-medically trained doctors would be treating patients who are accessing TCAM simultaneously. Therefore, there is need for doctors to be exposed to TCAM in their medical training. Research has found that bio-medical doctors in rural areas regularly encounter patients who visit traditional doctors initially before presenting themselves to bio-medical doctors [11,17]. The degree of interaction between TCAM and bio-medical modalities in the effective treatment of these patients is unknown.

## Methods

All eight South African medical schools were approached to participate although only seven consented. One was in KwaZulu-Natal, another in the Free State, one in Eastern Cape, two in Gauteng and two in the Western Cape.

Telephonic interviews were conducted with all available Heads of School followed by the submission of TCAM curricula to the researchers. Information on the description of the curriculum which included actual content and teaching duration was requested. All schools were asked to provide a detailed description of any individual courses dedicated to TCAM or courses where TCAM had been incorporated. This research further sought to identify at what level these courses were taught, whether at undergraduate or postgraduate level.

Ethical approval was obtained from the University of KwaZulu-Natal’s Biomedical Research Ethics Committee (Ethical Approval number: BF035/07).

## Results

Table 1 shows the level at which TCAM was being taught, with five schools teaching TCAM only at undergraduate level. One school was teaching an introductory course on TCAM at both an undergraduate and postgraduate level. In five schools TCAM was incorporated in other courses as a small component of the curricula to make students aware of its existence at under graduate level and at only one school it was included at postgraduate level. The telephonic interviews schedule is located in Additional file 1.

**Table 1:** Level of incorporation of TCAM into the curriculum (n = 7)

	**No. of medical schools**
TCAM at undergraduate level only	5
TCAM at undergraduate & postgraduate level	1
Not teaching TCAM at any level	1

One school stated it was not in their mandate to teach TCAM because they only taught material accredited by the Health Professionals Council of South Africa (HPCSA) who regulates bio-medical doctors.

Table 2 indicates that two schools had integrated Traditional African Medicine, with four schools teaching both Traditional African Medicine and CAM. None of the seven surveyed medical schools taught TCAM as an independent course within their medical programs.

**Table 2:** Characteristics of courses with TCAM (n = 7)

	**No. of medical schools**
Teaching Traditional African Medicine only	2
Teaching Traditional African Medicine and CAM combined	4
Not teaching Traditional African Medicine or CAM	1

Table 3 indicates those schools teaching TCAM practically and those schools teaching the concepts of TCAM only. Two schools were using the problem-based learning approach. This approach involves students learning through the experience of practical problem solving by exposing students to traditional healers. Four schools were only teaching the concepts of TCAM and did not have practical sessions for their students with traditional healers or CAM practitioners.

**Table 3:** Approach to teaching TCAM (n = 7)

	**No. of medical schools**
Problem-based learning approach	2
Only theory	4
Not teaching TCAM	1

Table 4 indicates the TCAM treatment practices schools were teaching. Out of Traditional African Medicine and the 11 TCAM treatment practices, schools were only teaching Traditional African Medicine and three CAM practices (Ayurvedic, Chinese Medicine and Homeopathy). Most schools (6 out of 7) confirmed teaching Traditional African Medicine and the content included lectures from traditional healers about African cosmology, as well as discussions about medicinal plant usage for certain illnesses. Only the medical school in KwaZulu-Natal taught more than one CAM practices (Chinese and Ayurvedic) and TM. All schools lacked a course specifically focusing on CAM only. In most instances the curriculum alluded to CAM practices in some courses at either undergraduate or postgraduate level training. However, the practices were not taught in depth but introduced to make students aware of them.

**Table 4:** TCAM practices taught (n = 7)

	**No. of medical schools**
Traditional African Medicine	6
Ayurvedic	1
Chinese Medicine	1
Homeopathy	1

## Discussion

This study investigated the extent to which South African medical schools have incorporated TCAM into their curricula. A lack of commitment to teaching TCAM was observed at almost all schools except one. This is despite the fact that bio-medically trained doctor’s practice in a context where patients consult TCAM practitioners as well as themselves [18]. Medical schools are not doing enough to ensure TCAM is covered within their curricula. At most of the medical schools TCAM was covered but in a tokenistic manner. The university in KwaZulu-Natal is the one exception.

Hollenberg [6] stressed TM was continuously being neglected by medical schools despite evidence of its significant influence within African countries. This observation is congruent with research conducted in Japan where only 20% of that countries medical schools offered CAM despite its widespread usage by the population. Interestingly, in Korea and Canada there was a contrary result. 85% of Korean and 77% of Canadian medical schools taught CAM [8]. According to Kim [8] TCAM had increased in popularity due to the fact of increased demand from patients and therefore students were more enthusiastic to learn about TCAM.

The challenge of incorporating TCAM into bio-medical curriculums was noted by Kim [8] who suggested that introducing new content faces many barriers including “a lack of an evidence-based approach, insufficiently reliable reference resources, and insufficient time to incorporate new courses”. Medical schools are therefore often resistance to fully incorporate TCAM due to curriculum overload or lack of adequate resources for its incorporation [7]. Another reason for the lack of inclusion of TCAM can be linked to the curriculum being biased towards an epistemology which undermines the value of indigenous knowledge systems whilst hailing biomedicine and western science [11].

Only two schools were teaching TCAM using a problem-based learning approach whilst four were teaching TCAM concepts only. Various teaching methods have been used to teach TCAM [8]. Practice sessions are not often used as a mode of teaching TCAM [8]. Only one medical school taught traditional medicine practically. More needs to be done to have practical sessions and increase the number of courses referring to TCAM. Barrows and Tamblyn [19] stress that when teaching health subjects, a problem-based approach is optimal as students are provided an opportunity gain practical experience through problem-solving. They further stress that problem solving skills are the most important skills to acquire rather than memory skill only [19].

There are no studies that have focused on investigating the integration TCAM into the undergraduate and postgraduate medical curricula of South African medical schools. Implications of our study include the need for further research on how medical schools are addressing the issue of potential drug interactions when patients use TCAM and bio-medicine simultaneously, and adherence in the curricula.

## Conclusions

There remains insufficient commitment by South African medical schools to incorporate TCAM into their curriculum. Most schools refer briefly to a few TCAM practices within their curriculum. Medical schools need to ensure TCAM has a more prominent role in their curricula considering the social, political and policy context of South Africa. There is need to acknowledge the reality that there is a constant interaction between bio-medical and TCAM treatment practices. Exposing doctors to TCAM will assist them to understand the paradigm from which TCAM practitioners treat patients. This exposure will hopefully lead to a harmonization of treatment methods with the aim of improving patient health outcomes. Therefore, there is an immediate need for all medical schools in South Africa to examine their curriculum with respect to TCAM and standardise the TCAM content across all universities.

## Competing interests

The authors declare that they have no competing interests.

## Authors’ contributions

GG and JG designed the study. GG interpreted the analysis and planned the manuscript. EC undertook data collection and analysis and contributed to writing the manuscript. All authors wrote, read and approved the final manuscript.

## Pre-publication history

The pre-publication history for this paper can be accessed here:

http://www.biomedcentral.com/1472-6920/14/40/prepub

## Supplementary Material

Additional file 1 Traditional complimentary & alternative medicine telephonic interview schedule. Click here for file

## References

[B1] Barnes PM Powell-Griner E McFann K Nahin RL Complementary and alternative medicine use among adults: United States, 2002 Sem Int Med 2004 2 54 71 10.1016/j.sigm.2004.07.003 15188733

[B2] Mak JC Faux S Complementary and alternative medicine use by osteoporotic patients in Australia (CAMEO-a): a prospective study J Altern Complement Med 2009 16 579 584 20491514 10.1089/acm.2009.0425

[B3] Singh V Raidoo DM Harries CS The prevalence, patterns of usage and people’s attitude towards complementary and alternative medicine (CAM) among the Indian community in Chatsworth, South Africa BMC Compliment Alt Med 2004 4 3 7 10.1186/1472-6882-4-3 PMC356921 15018622

[B4] Oh B Buttow P Mullan B Beale P Pavlakis N Rosenthal D Clarke S The use and perceived benefits resulting from the use of complementary and alternative medicine by cancer patients in Australia Asia Pac J Clin Oncol 2010 6 342 349 10.1111/j.1743-7563.2010.01329.x 21114784

[B5] World Health Organization Factsheet 134: traditional medicine 2013 Geneva: World Health Organization http://www.who.int/mediacentre/factsheets/2003/fs134/en/ Accessed July 1, 2013

[B6] Hollenberg D Zakus D Cook T Wei X Re-positioning the role of traditional, complementary and alternative medicine as essential health knowledge in global health: do they still have a role to play? World Health Popul 2008 10 62 75 19550163

[B7] Gqaleni N Moodley I Kruger H Ntuli A McLeod H Traditional and complementary medicine South Africa Health Rev 2007 12 175 188

[B8] Kim DY Park WB Kang HC Kim MJ Park KH Min B Suh D Lee HW Jung SP Chun M Lee SN Complementary and alternative medicine in the undergraduate medical curriculum: a survey of Korean medical schools J Altern Complement Med 2012 18 870 874 10.1089/acm.2011.0179 22849549

[B9] Labhardt ND Aboa SM Manga E Bensing JM Langewitz W Bridging the gap: how traditional healers interact with their patients. A comparative study in Cameroon Trop Med Int Health 2010 15 1099 1108 20545920 10.1111/j.1365-3156.2010.02575.x

[B10] Nahin RL Barnes PM Stussman BJ Bloom B Costs of complementary and alternative medicine (CAM) and frequency of visits to CAM practitioners: United States, 2007 National health statistics reports, No 18 2008 Hyattsville, MD: National Center for Health Statistics 19771719

[B11] Nxumalo N Alaba O Harris B Chersich M Goudge J Utilization of traditional healers in south africa and costs to patients: findings from a national household survey J Public Health Policy 2011 32 124 136 10.1057/jphp.2011.26 21730986

[B12] Peltzer K Preez NF Ramalagan S Fomundam H Use of traditional complementary and alternative medicine for HIV patients in KwaZulu-Natal, South Africa BMC Public Health 2008 8 255 10.1186/1471-2458-8-255 18652666 PMC2503977

[B13] Dantley ME Uprooting and replacing positivism, the melting pot, multiculturalism, and other impotent notions in educational leadership through an African american perspective Educ Urban Soc 2002 34 334 352 10.1177/0013124502034003004

[B14] Fenton MV Morris DL The integration of holistic nursing practices and complementary and alternative modalities into curricula of schools of nursing Alt Therap Health Med 2003 9 62 67 12868254

[B15] Parliament of South Africa Traditional health practitioners Act Act No 22 of 2007, parliament of south africa, cape town, government gazette 2007

[B16] Nick L South Africa shows support for rural medicine Lancet 1995 346 344

[B17] Reardon C George G Jimmyns C The Mobility of Health professionals 2011 Geneva: Final Report. International Organization for Migration

[B18] Mngqundaniso N Peltzer K Traditional healers and nurses: a qualitative study on their role on sexually transmitted infections including HIV and AIDS in KwaZulu-Natal, South Africa Africa J Trad Complement Alt Med 2008 5 380 386 10.4314/ajtcam.v5i4.31293 PMC2816575 20161960

[B19] Barrows HS Tamblyn RM Problem based learning: an approach to medical education 1980 New York: Springer Publishing Company

