# Supplementary material for: A review of the integration of traditional, complementary and alternative medicine into the curriculum of South African medical schools
Source: BMC Med Educ. 2014 Feb 28;14:40. doi: 10.1186/1472-6920-14-40 (PMC3939811; doi:10.1186/1472-6920-14-40)
Supplement: Additional file 1 — Traditional complimentary & alternative medicine telephonic interview schedule. [file 1472-6920-14-40-S1.docx]

**Traditional Complimentary & Alternative Medicine Telephonic Interview Schedule**

University Name:

Province located:

Name of Head of Department:

1. **Which courses cover traditional complimentary/alternative medicine (TCAM) at your university?**
2. **Are there dedicated TCAM courses and in which academic year are they offered?**
3. **Please provide a detailed description of the course content**
4. **Please provide us with assessment task examples**

1. **Please provide details on teaching methods adopted with each of the TCAM courses and the allocated student contact time for each course**
2. **If a problem based learning approach is used, please provide examples of how you make use of it**
